# PARP-1-Associated Pathological Processes: Inhibition by Natural Polyphenols

**DOI:** 10.3390/ijms222111441

**Published:** 2021-10-23

**Authors:** Natalya V. Maluchenko, Alexey V. Feofanov, Vasily M. Studitsky

**Affiliations:** 1 Biology Faculty, Lomonosov Moscow State University, Lenin Hills 1/12, 119234 Moscow, Russia; avfeofanov@yandex.ru (A.V.F.); vasily.studitsky@fccc.edu (V.M.S.); 2 Shemyakin-Ovchinnikov Institute of Bioorganic Chemistry, Russian Academy of Sciences, Mikluko-Maklaya Str., 16/10, 117997 Moscow, Russia; 3 Fox Chase Cancer Center, Cottman Avenue 333, Philadelphia, PA 19111, USA

**Keywords:** PARP-1, polyphenols, PARP-1 inhibitors

## Abstract

Poly (ADP-ribose) polymerase-1 (PARP-1) is a nuclear enzyme involved in processes of cell cycle regulation, DNA repair, transcription, and replication. Hyperactivity of PARP-1 induced by changes in cell homeostasis promotes development of chronic pathological processes leading to cell death during various metabolic disorders, cardiovascular and neurodegenerative diseases. In contrast, tumor growth is accompanied by a moderate activation of PARP-1 that supports survival of tumor cells due to enhancement of DNA lesion repair and resistance to therapy by DNA damaging agents. That is why PARP inhibitors (PARPi) are promising agents for the therapy of tumor and metabolic diseases. A PARPi family is rapidly growing partly due to natural polyphenols discovered among plant secondary metabolites. This review describes mechanisms of PARP-1 participation in the development of various pathologies, analyzes multiple PARP-dependent pathways of cell degeneration and death, and discusses representative plant polyphenols, which can inhibit PARP-1 directly or suppress unwanted PARP-dependent cellular processes.

## 1. Introduction

Poly (ADP-ribose) polymerase-1 (PARP-1) is a widespread nuclear protein with a spectrum of different activities due to its DNA-binding and enzymatic properties [1,2,3,4,5]. PARP-1 uses β-NAD+ as a substrate to synthesize branched polymers of ADP-ribose (PAR) and to covalently modifies more than 40 nuclear proteins and transcription factors, including PARP-1 itself. Under conditions of moderate genotoxic stress, the functioning of PARP-1 maintains integrity and activity of cell genome, while during severe genotoxic stress PARP-1 coordinates multiple pathways of cell death. General enhancement of PARP-1 activity is associated with the development of tumor, cardiovascular and neurodegenerative diseases, and pharmacological inhibition of PARP-1 is a promising strategy for their therapy. Several inhibitors of the enzymatic activity of PARP-1 (PARPi) are already used in clinical practice for the treatment of cancer [6,7]. A search for more active and less toxic PARPi, as well as for compounds that block the development of unwanted PARP-dependent cellular processes is in progress. In particular, a search for PARPi is carried out among natural compounds, since they might have a higher bioavailability, more effective cell penetration, higher pharmacological activity and fewer side effects than synthetic agents. Polyphenols are the largest and most studied group of plant metabolites, among which a considerable number of compounds were found to have therapeutic potential due to antiviral, antibacterial, antioxidant or antitumor activities. Some polyphenols were demonstrated to be effective PARPi or/and can affect signaling pathways that regulate cell survival under adverse conditions of oxidative/nitrosative or genotoxic stress. Many of these signaling pathways are closely related to molecular processes that are under the control of PARP-1. Accordingly, in the first part of the review, data are systematized on PARP-1-dependent molecular mechanisms that contribute to the development of diseases and therefore are targets for therapeutic intervention; in the second part, the polyphenols are discussed, which affect PARP-1 or (and) the signaling pathways under its control.

## 2. Relationship of PARP-1 with Inflammatory and Metabolic Diseases

**Figure 1:**
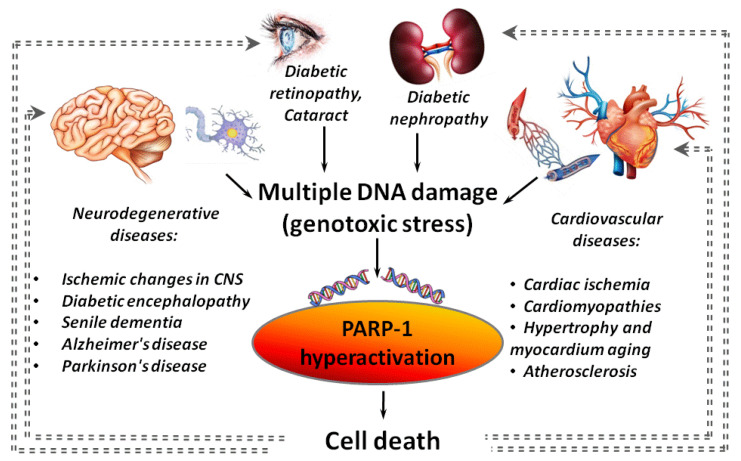
PARP-1 hyperactivation as an aggravating factor in the development of various diseases. Gray dotted lines indicate “vicious circles” when PARP-1 hyperactivation initiated by inflammation, cardiovascular, neurodegenerative or diabetic pathology leads to an increase in the severity of the disease.

Hyperactivation of PARP-1 plays an important role in the development of diseases, which are associated with or caused by chronic inflammation [8]. These include diabetes, neurodegenerative disorders (Alzheimer’s disease (AD), Parkinson’s disease (PD)), and cardiovascular diseases (Figure 1) [9,10,11,12].

Hyperactivation of PARP-1 was found in various cardiovascular diseases (ischemic heart disease, atherosclerosis, cardiomyopathies of various origins, hypertrophy and aging of the myocardium) [13,14] and in many models of a central nervous system (CNS) damage (stroke [15], traumatic brain injury [16], neurodegeneration [17] and senile dementia [18]). A negative role of the PARP-1 hyperactivation in cardiac ischemia/reperfusion is known for a long time [13,19]. It was shown that the strongest activation of PARP-1 is observed in a peri-infarction zone and areas of necrotic damage during a heart attack [20]. PARP-1 hyperactivation is involved in a cascade of events initiated by β-amyloid peptides (Aβ), the accumulation of which leads to the death of brain cells in AD. A significant increase in PARP-1 expression and accumulation of PAR polymers was found in the cerebral cortex at the early (3.5 months) and intermediate (6 months) stages of Aβ-aggregation in mouse models of AD [21]. Hyperglycemia is also associated with PARP-1 hyperactivation [22,23,24] that is usually an aggravating factor in the development of systemic diabetic dysfunctions. In particular, PARP-1 hyperactivation is involved in the death of insulin-producing pancreatic β-cells [25].

**Figure 2:**
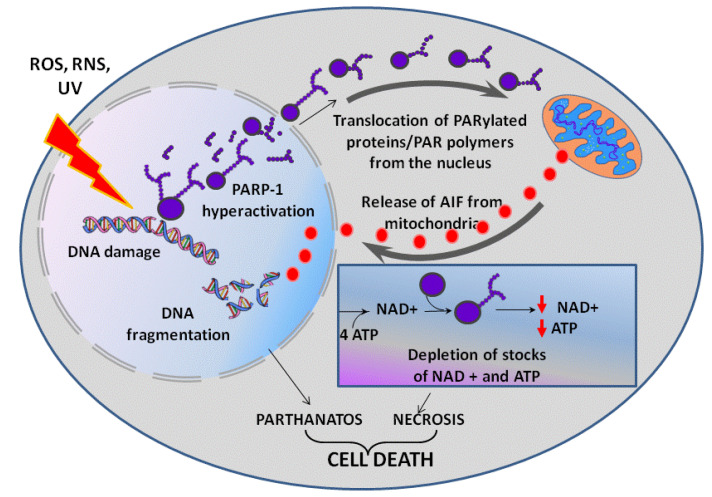
PARP-1 dependent cell death. UV—ultraviolet light, ROS—reactive oxygen species overproduced in oxidative stress, RNS—reactive nitrogen species (e.g., nitric oxide NO) overproduced in nitrosative stress. See text for detail.

Inflammatory processes, hypoxia, hypo- and hyperglycemia are often accompanied by an uncontrolled increase in the levels of reactive oxygen (ROS) and nitrogen species (RNS), which cause DNA damage. As a consequence, an increase in PARP-1 activity is required for DNA repair. In turn, PARP-1 hyperactivation can initiate parthanatos—a programmed caspase-independent cell death (Figure 2) [26,27]. PAR and PARylated proteins that are formed in large quantities migrate from a nucleus to cytoplasm and cause the release of apoptosis-inducing factor (AIF) from mitochondria [26,28,29]. Released AIF is transported to a nucleus due to a nuclear localization signal (NLS) and activates endonucleases, which cause DNA fragmentation followed by cell death. PARP-1-mediated parthanatos is observed in neurons during PD, excitotoxicity of glutamate and cerebral ischemia [30,31].

The PARP-1-dependent cell death can also occur after PARP-1 hyperactivation due to an energy crisis caused by the depletion of cellular reserves of macroergic compounds [32,33]. A synthesis of a NAD+ molecule requires four ATP molecules, and intense consumption of NAD+ by PARP-1 can result in a rapid depletion of ATP and NAD+ stocks, lead to suppression of energy-dependent cellular processes followed by cell necrosis [34]. Suppression of energy-dependent processes is additionally enhanced by PAR metabolism. Free and protein-bound PAR is intensely cleaved by poly(ADP-ribose)glycohydrolase to ADP-ribose, which is then metabolized by NUDIX-hydrolases (NUDIX - NUcleoside DIphosphates linked to any other moiety X) to AMP [35]. A high AMP/ATP ratio is interpreted by a cell as an energy stress, and AMP-activated protein kinase corrects this apparent energy misbalance by blocking the mammalian target of rapamycin (mTOR) signaling pathway with a subsequent down-regulation of ATP consuming processes [36].

**Figure 3:**
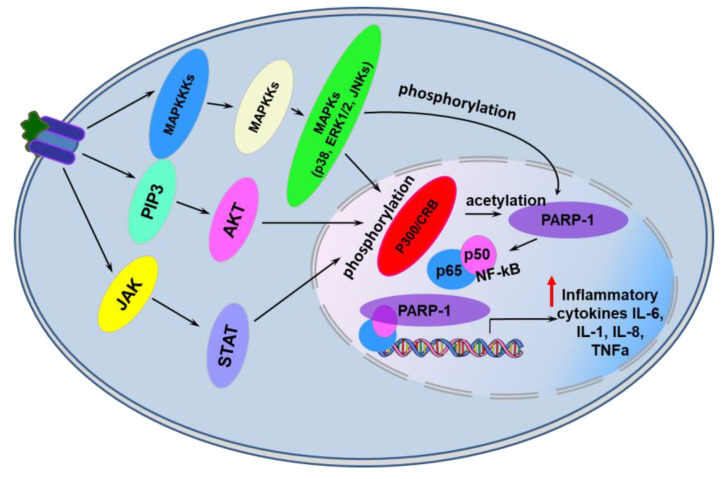
PARP-1-dependent transcriptional activation of genes encoding pro-inflammatory cytokines in eukaryotic cells. See text for details. Abbreviations: JAK—Janus kinase; PIP3—phosphatidylinositol (3,4,5)-trisphosphate; MAPKKKs—Mitogen-Activated Protein (MAP) kinase kinase kinases; MAPKKs—Mitogen-activated protein kinase kinases; MAPKs—mitogen-activated protein kinases; STAT—members of the signal transducer and activator of transcription protein family; AKT—subfamily of serine/threonine kinases; p38—p38 mitogen-activated protein kinases; JNK—c-Jun N-terminal kinases; ERKs—extracellular signal-regulated kinases; IL-l, IL6, IL-8—interleukins 1, 6, 8, p300/CRB—p300/CREB-binding protein complex, TNFα—tumor necrosis factor α.

PARP-1 itself promotes the development of inflammatory processes by up-regulating expression of various inflammatory mediators such as tumor necrosis factor α (TNFα), inducible isoform of nitrite oxide synthase (iNOS), cyclooxygenase 2 (COX2), monocyte chemoattractant protein 1 (MCP1), interleukins 1β and 6 (IL-1β, IL-6). Here PARP-1 acts as a co-activator of transcription factors such as nuclear factor kappa-light-chain-enhancer of activated B cells (NF-κB), activator proteins 1 and 2 (AP1, AP2) that regulate immune and inflammatory responses (Figure 3) [37,38,39,40]. PARP-1 was shown to be acetylated at lysine residues (K498, K505, K508, K521, K524) by the p300/CREB-binding protein complex (CREB - cAMP-response element binding protein) and phosphorylated at Y829 by mitogen-activated protein kinases (MAPKs) in response to pro-inflammatory stimuli [37,38]. Modified in this way, PARP-1 stimulates transcription of NF-κB-dependent genes of inflammatory mediators (Figure 3) [37,38,39,40]. Interestingly, neither the enzymatic activity of PARP-1 nor its DNA-binding activity were required for full activation of NF-kB in response to various stimuli [37]. PAR polymers can act as alarmins releasing from a cell during stress and activating production of inflammatory cytokines by the cells of an innate immunity system [41].

During apoptosis, which may accompany the development of some pathologies, PARP-1 is cleaved by caspases 3 and 7 into DNA-binding and catalytically active fragments [42,43], but retains its ability to activate NF-κB and enhance transcription of inflammatory mediator genes [44]. The C-terminal fragment preserves catalytic activity, but is not stimulated by DNA damage. The N-terminal fragment remains associated with DNA injuries blocking access of repair factors to them [45,46].

As a co-activator of NF-κB and activator protein 1, PARP-1 was suggested to be responsible for accelerated aging during chronic inflammatory diseases [47].

An important role of PARP-1 in the development of inflammatory diseases was confirmed by experiments with PARP-1 knockout mice. These mice are better protected from diabetic and septic complications associated with inflammation such as multiple organ dysfunction syndromes [37,48].

In summary, PARP-1 hyperactivation, which occurs during oxidative/nitrosative stress, chronic inflammation and irreversible genotoxic damage, leads to massive cell death that at the level of the organism promotes development of metabolic syndrome, multiple organ dysfunction syndrome, cardiovascular and neurodegenerative diseases.

## 3. PARP-1 and Oncological Diseases

PARP-1 is involved in pathogenesis of oncological diseases in a complex way as described in several excellent comprehensive reviews [49,50,51,52]. Here we will only briefly describe the effect of PARP-1 on tumor cell metabolism, referring readers to the published reviews for more details.

In contrast to the negative role of severe hyperactivation of PARP-1 in inflammatory processes, a moderate activation of PARP-1 occurring during transformation of cells does not lead to a cell death. On the contrary, it contributes to cell survival. The pro-tumor activity of PARP-1 is mediated by PARP-1-dependent deregulation of factors involved in the cell cycle, mitosis, apoptosis and autophagy [53]. PARP-1 impedes with cell differentiation thus enhancing tumor malignancy [54], and moderate activation of PARP-1 caused by the accumulation of DNA damage during intensive cell division increases DNA repair efficiency and cell viability.

Malignancy of cancer cells and its ability to metastasize strongly depend on the tumor microenvironment [55]. PARP-1 plays an important role in the functioning of the tumor microenvironment, participating in angiogenesis as well as in the formation of a tumor-associated stroma [56]. PARP-1 is involved in the process of epithelial-mesenchymal transition (EMT) during the acquisition of the ability of tumor cells to metastasize [52]. PARP-1 knockdown leads to EMT reversal through inhibitory transcription factors such as Smad4, p65 and ZEB1 [57].

**Figure 4:**
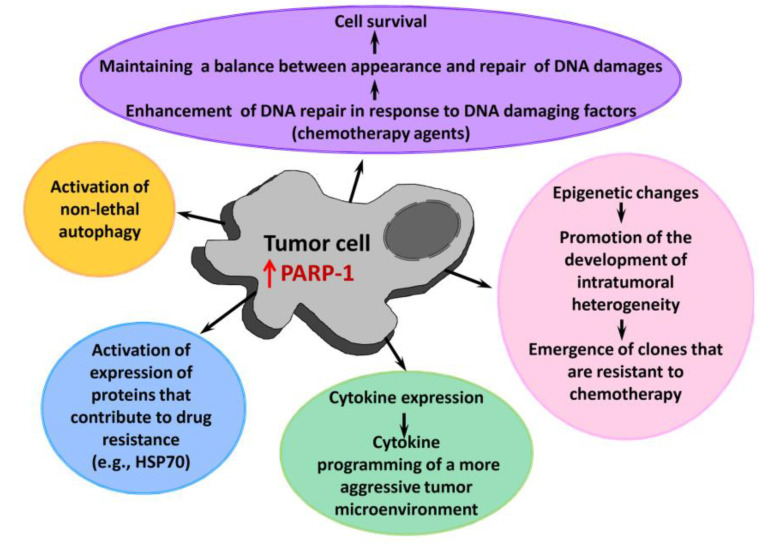
A role of PARP-1 in the tumor progression and development of its drug resistance. HSP70—heat shock protein 70.

PARP-1 participates in several processes responsible for the resistance of tumor cells to therapy (Figure 4) [25,30,58,59,60]. As a key repair enzyme, PARP-1 ensures the stability of a tumor cell genome after treatment with DNA damaging chemotherapy agents [61,62,63]. PARP-1 is able to promote (directly or indirectly) epigenetic modifications, creating conditions for development of heterogeneity of tumor cells and formation of super-resistant clones in a heterogeneous population [64]. Another PARP-1-mediated mechanism of drug resistance is a non-lethal autophagy [65,66,67]. PARP-1 is also known to control the expression of heat shock protein 70 [68,69], which makes a significant contribution to the survival of tumor cells and their resistance to antitumor agents [70].

In general, an increased level of PARP-1 expression is considered to be a prognostic marker associated with an aggressive phenotype of malignant tumors and a worse prognosis of patient survival [71,72,73].

## 4. Synthetic PARP-1 Inhibitors in Treatment of Diseases

**Figure 5:**
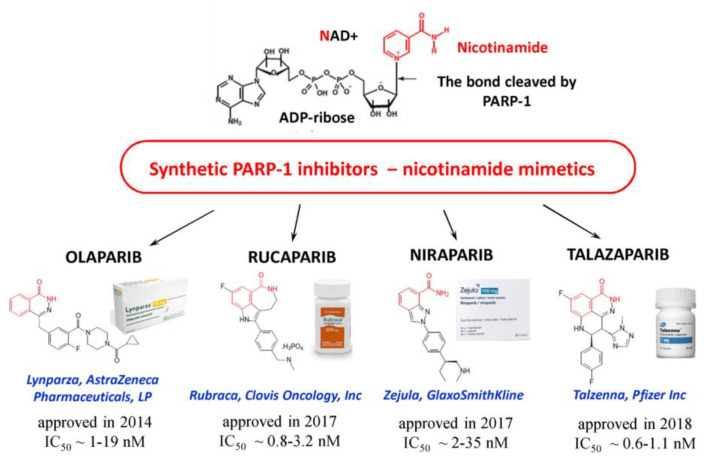
Synthetic PARPi approved for use in oncology. IC50 values (PARPi concentrations inducing 50% inhibition of PARP-1 activity) are cited from [19].

PARPi are considered to be promising antitumor agents since the increased activity of PARP-1 is a key factor contributing to growth of tumors, to an increase in their malignancy and to the development of drug resistance [74]. Most PARPi that are currently in antitumor preclinical and clinical trials are nicotinamide mimetics. They act by competing with NAD+ for the binding to a catalytic domain of PARP-1 and suppressing PAR synthesis [74]. Several PARPi are already used in clinical practice (Figure 5), and their combined administration with chemotherapy agents is promising for overcoming the drug resistance of tumor cells [75]. Inhibition of PARP1 is especially toxic to cells lacking functional forms of the tumor suppressors, breast cancer type 1 susceptibility protein (BRCA1) or breast cancer type 2 susceptibility protein (BRCA2) [76].

PARPi apparently can find wide application in the treatment of diseases related to inflammation. There are numerous examples of pharmacological or genetic inactivation of PARP-1 leading to a powerful anti-inflammatory effect that were demonstrated using different models of respiratory, gastrointestinal, osteochondral, cardiovascular and neurological pathologies (Table 1).

Importantly, PARPi block NF-κB-mediated transcription of genes encoding pro-inflammatory cytokines, but do not reduce the transcription of anti-inflammatory cytokine-encoding genes of IL-10 and IL-13 [99]. At the same time, even clinically approved PARPi are characterized by side effects that demand the search for safer drugs that target PARP-1 [100].

## 5. Polyphenols as PARP-1 Inhibitors

**Figure 6:**
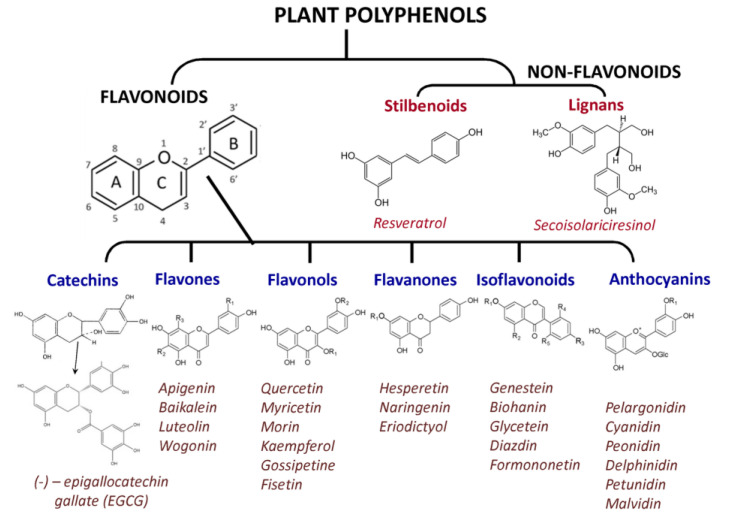
Plant polyphenols with known pharmacological properties.

An alternative to synthetic PARPi can be found among natural compounds—plant metabolites. Polyphenolic compounds (flavonoids and non-flavonoids, Figure 6), along with terpenoids and alkanoids, are the most common secondary plant metabolites. Flavonoids, which are subdivided into flavones, flavonols, flavanones, catechins (flavan-3-ols), isoflavonoids, and anthocyanidins (Figure 6) are the most widespread and studied natural polyphenols.

Various types of plant polyphenols have different anti-inflammatory, antioxidant, anti-allergic, antiviral and/or antitumor activities [101,102]. Some natural polyphenols are epigenetically active compounds and may play an important role in the regulation of gene expression, including PARP-dependent genes [103]. Some of polyphenols were shown to have PARP-1 inhibiting activity [104,105,106]. Many polyphenols have high bioavailability, efficiently penetrate cells and induce biological effects at micromolar concentrations [107,108,109,110] that makes them good candidates for the search of new PARPi. Thus, a search in the flavonoid library led to the discovery of PARPi such as myricetin, quercetin, fisetin, tricetin, gossipetin and delphinidin [104]. Functional screening of the library of polyphenols used in traditional medicine resulted in identification of 11 compounds interacting with PARP-1 with the dissociation constants of the complexes ranging from 0.32 to 79 µM [111]. The most active PARPi among the polyphenols was 2”-hydroxygenkwanol A isolated from the plant *Daphne linearifolia* that has long been used to treat inflammation and rheumatism in Arab traditional medicine. This polyphenol is structurally similar to talazaparib, the strongest synthetic PARPi. Computer screening technologies also predict an existence of PARPi among polyphenols that may have affinities higher than clinically approved synthetic PARPi [112].

Below the features of PARP-1 inhibition by some representatives of polyphenols are considered in more detail.

### 5.1. Flavonols 

Flavonols are the most abundant species of flavonoids existing in nature. Their distinct feature is the presence of 3-hydroxyflavone in the structure. Flavonols are often found as O-glycoside, glucuronide, methyl, and sulfate conjugates.

The flavonol quercetin (QC) is found in large quantities in plants, predominantly red and orange (sea buckthorn, cranberries, raspberries, blueberries, onions), as well as in food products such as buckwheat, tea, red wine, and olive oil. QC is usually found in plants in conjunction with glycosylated forms—isoquercetin and rutin [113]. These flavonols perform a wide range of physiological functions in plants, the most important of which is antioxidant. QC is able to inhibit PARP-1 in the micromolar concentration range, and its activity is approximately seven times higher than that of 3-aminobenzamide (3-AB) [114]. It was found that glycosylation improves the solubility of QC derivatives, but decreases their inhibitory activity. It was shown that QC at a concentration of more than 30 µM exhibits genotoxicity. Glycosylated analogs have less cyto- and genotoxicity, but this might be due to their lower cell permeability.

Regular consumption of citrus fruit reduces the risk of cancer, and this effect is likely associated with inhibition of PARP-1 by flavonols naringenin (NG), hesperetin (GP) and their O-glycoside forms naringin and hesperidin, which are contained in citrus fruit [115]. GP was found to be more active than QC and cytotoxic for both wild-type V79 cells and mutant cells deficient in the BRCA2 protein involved in DNA repair (100% cytotoxicity at 30 µM GP) [116]. In turn, QC selectively induces the death of BRCA2 mutant cells (40% cytotoxicity at 30 µM QC).

Glycosylated isoquercetin, rutin, naringin, and hesperidin have less cytotoxicity than the corresponding non-glycosylated flavonoids, but at the same time exhibit selectivity towards BRCA2 mutant cells [115]. The death of more than 80% of the mutant cells was observed at 100 µM rutin and isoquercetin and at ~1 mM naringin and hesperidin.

In hepatocytes stimulated by the pro-inflammatory cytokine IL-1, QC reduces NO production through suppression of iNOS expression, which in turn, can block the enhancement of the inflammatory cascade [116]. Similarly, QC inhibits the LPS-induced iNOS gene expression in various models [117,118,119]. It is believed that the anti-inflammatory effect of QC is based on a combination of antioxidant and anti-PARP activities. Other flavonoids such as naringenin, apigenin, and resveratrol also block iNOS expression in macrophages [120,121].

Flavonoids are able to attenuate NAD+ depletion by inhibiting PARP-1 hyperactivation both in vitro and in vivo [104], therefore reducing the likelihood of cell death and exerting a pleiotropic protective effect at high glucose levels [122]. This may play an important role in preventing the development of diabetic complications caused by increased PARP-1 activity, including those associated with massive neuronal death [123]. The molecular mechanisms of the protective action of flavonoids in the suppression of diabetic complications remain the subject of active study [124]. QC can up-regulate expression of neural and synapse-associated proteins (nerve growth factor, brain-derived neurotrophic factor, post synaptic density 93 and 95 proteins) and inhibit neurodegeneration [125]. QC increases the level of SIRT1 (NAD+-dependent histone deacetylase) and inhibits the stress proteins of the endoplasmic reticulum (RNA-like endoplasmic reticulum kinase, inositol-requiring enzyme-1α, activating transcription factor 6α, eukaryotic initiation factor 2, binding immunoglobulin protein and protein disulfide isomerase). An increase in SIRT1 activity was shown to have a positive effect on the metabolism of mammals, leading to inhibition of aging and longevity [126,127]. PARP-1 knockout increases the NAD + content and, accordingly, SIRT1 activity in brown adipose tissue and muscles [128]. A similar effect is caused by PARPi. In aging rats, QC activates SIRT1, promotes monoamine synthesis and improves animal cognitive functions. QC improves learning and memory in diabetic rats [124,129,130].

### 5.2. Flavones

Flavones are flavonoids that have a 2-phenylchromen-4-one (2-phenyl-1-benzopyran-4-one) group. 4’-Methoxyflavone (4MF) and 3’, 4’-dimethoxyflavone (DMF) were reported to prevent parthanatos in cells treated with a DNA-alkylating agent and possess neuroprotective activity [131]. It was concluded that the anti-parthanatos effect of 4MF and DMF is related to the suppression of PARP-1 activity and is not associated with antioxidant or free radical scavenging properties. Both compounds almost equally prevented parthanatos in HeLa cells, but 4MF demonstrated higher neuroprotection than DMF.

It should be noted that some flavones (e.g., apigenin and luteolin) inhibit tankyrases (TNK), the proteins of the PARP family, which are attractive targets in cancer treatment [132]. The antitumor therapeutic potential of TNK is determined by their participation in telomere homeostasis, mitosis, and Wnt signaling pathways [133]. Interestingly, flavones do not contain the nicotinamide-like moiety that is characteristic for most PARP-1 inhibitors, and the flavone-based pharmacophore model was designed for TNK inhibitors [134,135].

### 5.3. Catechins

Flavan derivatives catechins include a wide variety of biologically active compounds. A feature of their structure is the absence of a double bond between the second and third carbon atoms leading to emergence of two chiral centers and four diastereoisomers. The trans and cis isomers are called catechins and epicatechins, respectively. Catechins are present in large quantities in tea leaves and cocoa beans. Green tea contains epigallocatechin gallate (EGCG), which is considered one of the most powerful dietary antioxidants [136]. During the production of black tea (enzymatic oxidation), catechin is oxidized to quinone, which is further condensed into several other chemical compounds, one of which is the theaflavin polyphenol (TF). Tea polyphenols affect regulatory systems of cells and may produce an inhibitory effect on various stages of carcinogenesis: inflammatory processes, cell transformation, proliferation, apoptosis, metastasis, and invasion [107,137,138,139,140]. It was found that EGCG and TF cause synthetic lethality in BRCA2-deficient cells through a PARP-dependent mechanism [141]. EGCG inhibits PARP-1 more effectively than TF, which is probably due to the presence of a haloyl group. Moreover PARP-1, the targets of tea polyphenols are histone deacetylases [142], transcription factors [143], DNA topoisomerase II [109] and ABC transporters responsible for the development of multidrug resistance [144,145].

Other catechins that affect PARP-1 include epicatechin, myricetin, epigallocatechin, catechin gallate, epicatechin gallate, gallocatechin, and gallocatechin gallate [146].

### 5.4. Resveratrol

The representative of stilbenoids, resveratrol (RSV), exists in the form of cis- and trans-stereoisomers and is often glycosylated. RSV is found in grapes, nuts and cocoa beans, as well as in berries, leaves and flowers of orchids, eucalyptus, gnetum and some other plants. Numerous studies have shown that the RSV containing extracts reduce thrombus formation, improve the rheological properties of blood, relax the vascular endothelium, lower cholesterol and triglyceride levels in the blood preventing atherosclerosis development, exhibit antioxidant and anti-inflammatory activity [147,148,149]. Such properties are strongly associated with the RSV-mediated blocking of the mTOR (mammalian target of rapamycin) signaling pathway [150]. mTOR is known to integrate various signaling pathways, including the pathways of insulin, growth factors, and mitogens. It functions as a sensor for redox status and cellular nutrient and energy levels. Dysregulation of mTOR pathway leads to the development of various metabolic and oncological diseases. The mTOR pathway can intersect with PARP-1 during partanotosis. In this case, SIRT1, involved in the regulation of the intracellular level of NAD +, can play an important role as a factor that binds PARP-1 and mTOR pathway [151]. It was demonstrated that RSV directly binds to PARP-1 and induces its dose-dependent inhibition (IC50 = 0.65 μM) [152]. Treatment of cells damaged by hyperglycemia with RSV reduces the production of ROS, improves the ratio of reduced/oxidized glutathione (GSH/GSSG), restoration mitochondrial membrane potential [153]. Studies on the suppression of metabolic stress leading to the onset of diabetic cataracts revealed that the administration of RSV led to a significant decrease in cataractogenesis. This effect may be associated with both the activation of antioxidant protection and the inhibition of PARP-1.

## 6. Conclusions

A moderate level of PARP-1 activation provides an effective reparation of DNA lesions supporting survival of cells under the action of genotoxic factors. A hyperactivation of PARP-1, which often occurs at the inflammation, hypoxia, hypo- and hyperglycemia, modulates or activates multiple cellular pathways leading to cell death or degeneration. Synthetic PARPi are already implemented in anticancer therapy, and might also be useful in treatment of metabolic syndrome, multiple organ dysfunction syndrome, diabetic complications, cardiovascular and neurodegenerative diseases.

**Figure 7:**
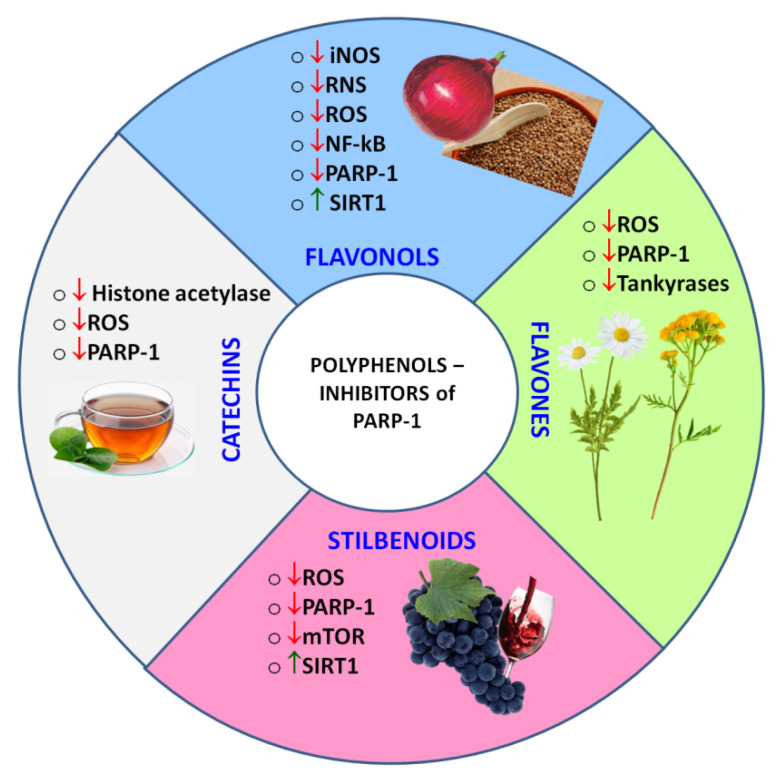
Classes of polyphenols, whose representatives were found to act as PARPi, and the observed polyphenol-induced regulatory effects. ↑ - up-regulated, ↓- down-regulated.

Natural polyphenols capable of inhibiting PARP-1 directly or indirectly (Figure 7) can become a supplement or even an alternative to synthetic drugs, because besides a pronounced pharmacological activity they could have low systemic toxicity and minor side effects. As an adjunct to standard drug therapy, polyphenols can allow one to reduce a concentration of toxic drugs, providing a synergistic effect.

Extending a search for natural PARPi among the secondary plant metabolites, terpenoids should be also considered. Terpenoids, like polyphenols, have a wide spectrum of biological activities [154,155] and some of them were reported to be PARPi [21,140].

## Figures and Tables

**Table 1:** Inhibition of PARP-1 in the treatment of non-cancer diseases.

Targeted Organ/System	Model	PARP-1 Inhibition Method	Effects	Ref.
The cardiovascular system	Various models of myocardial ischemia/reperfusion, models of acute coronary syndrome in mice and rats, as well as atherosclerotic vascular lesions	Genetic suppression (hereinafter—PARP-1^−/−^ or activity inhibition with PARPi: 3-AB, TIQ-A, PJ-34, ABT-888, DPQ, INO-1001 or Doxycycline	↑TIMP-2, ↓NF-κB, ↓MCP-1, ↓ICAM-1, ↓TNF-α, ↓nitrotyrosine (marker of NO-dependent oxidative stress), ↓attraction of macrophages (MF), ↑ALDH2, ↓TC, ↓VLDL, ↓LDL, ↓ACAT-1, ↓caspase-3, ↑SMCs and collagen content, ↓atherosclerotic plaques, ↓MMPs, ↓ infarction zone, ↓CRP, ↓IL-6, ↓MPO activity, ↓neutrophil infiltration, ↓iNOS, ↓AIF nuclear translocation	[13]
Lungs, liver, gut, CNS	LPS-induced sepsis and endotoxic shock in animal model (mice, mini-pigs, rats)	PARP-1^−/−^or PARPi: PJ34, Olaparib, 3-AB or INO-1001	↓degree of organ inflammation ↓TNF-α, ↓IFN-γ, ↓iNOS, ↓IL-1β, ↓IL-6, ↑IL-10, ↓neutrophil infiltration, ↓increased vascular permeability in organs, ↓NO production, ↓lipid peroxidation, ↓MIP-1α↓MIP-2 (CXCL2), ↓MCP-1, ↓CXCL1 (mKC), ↑protective effect on membrane lipids	[40,77,78,79]
Gastrointestinal tract	Salmonella-induced sepsis in animal model (mice)	PARP-1^−/−^	↓CXCL9, ↓Gbp2, CXCL10, ↓Iigp1, Cd274, ↓IFN-γ,	[80]
Gastrointestinal tract	TNBS-induced colitis in animal model (mice)	PARP-1^−/−^	↓ICAM-1, ↓neutrophil infiltration, ↓lipid peroxidation,↓degree of nitrosative lesion.	[81]
CNS	Induced stroke in animal model (primates, mice, rats)	PARP-1^−/−^or PARPi: benzamide, 3-AB, ISQ, DPQ, PHT, INH2BP, GPI-6150, PJ34, INO-10001, ONO-1924H, DR2313, GPH, MP-124 or JPI-289	↓PARP-1 activity, ↓PAR in the affected area, ↓inflammation and swelling of the brain,↓secondary neuronal damage	[82,83,84,85]
CNS	Alzheimer’s disease: in vitro cellular models (human and rat cells treated with Aβ peptide); in vivo animal models (mice, rats with Aβ peptide)	PARPi: benzamide, Rukaparib, Veliparib, MC2050, PJ34, INO-1001, JPI-289, nicotinamide	↓neuroinflammation, ↓accumulation of Aβ plaques, ↑genes of antioxidant defense enzymes (*Sod1, Gpx1, Gpx4*), ↑genes regulating the mitochondriongenesis (*Mfn1, Mfn2, Dnm1l, Opa1, Fis1*), ↑mt-Nd1, ↑Sdha, ↑mt-Cytb, ↓membrane potential of mitochondria,↑Foxo1, ↓Nrf1, ↓Stat6, ↓NF-κB, ↓free radical concentration	[86,87,88,89]
CNS	Parkinson’s disease: 6-OHDA-induced mice model of PD, MPTP-induced dopamine neurotoxicity, AIMP2 transgenic mice.	PARP-1^−/−^ or PARPi: benzamide or Rukaparib	↓atrophy of dopaminergic (DA) neurons; ↓degeneration of DA neurons	[90,91,92]
Spinal cord	Spinal cord injuries in mice	PARPi: 3-AB or 5-AIQ	↓infiltration of the injured spinal cord with neutrophils, ↓cell apoptosis, ↓spinal cord injury	[93]
Diabetic multiple organ lesions	Mice, rats (by high-fat feeding and a single peritoneal dose of streptozotocin or obese animals with leptin resistant	PARPi: INO1001, MRL-45696 or JPI-289	↑SIRT1, ↑PGC-1α, ↓oxidative stress, ↓organ inflammation and fibrosis,↓TLR4, ↓NFκB signaling pathway.	[13,94,95,96]
Immune system	Arthritis in mice or rats	PARP-1^−/−^ or PARPi: 3-AB	↓IL-17, ↓TNF-α, ↓IL-2, ↓MCP-1, ↓MIP-2, ↓VCAM-1, ↓ICAM-1 at the site of defeat, ↓iNOS, ↓COX-2, ↓MMP-2, ↓MMP-9	[8,97,98]

Abbreviations: AMPK—AMP-activated protein kinase; TNBS—trinitrobenzenesulfonic acid; PGC-1α—peroxisome proliferator-activated receptor gamma coactivator 1-α; TIMP-2—tissue inhibitor of metalloproteinases 2, ICAM-1—inter-cellular adhesion molecule 1, ALDH2 -aldehyde dehydrogenase,2, TC—total cholesterol, VLDL—very low density lipoproteins, LDL—low density lipoproteins, ACAT1—acetyl-CoA acetyltransferase 1, SMC—smooth muscle cell content, CRP—C-reactive protein, MPO—myeloperoxidase, MMPs—matrix metalloproteinases, MIP-1a and 2—macrophage inflammatory proteins 1a and 2, CXCLs—C-X-C motif chemokine ligands, GBP2- guanylate binding protein 2, IigP1—interferon-inducible GTPase 1, CD274—programmed death-ligand 1 (or PD-L1), Gpx1,4—glutathione peroxidase 1, 4, SOD1—superoxide dismutase 1, mt-Nd1—mitochondrially encoded NADH, Sdha—succinate dehydrogenase complex flavoprotein subunit A, mt-Cytb—mitochondrially encoded cytochrome B, FOXO1—forkhead box protein O1, Nrf1—nuclear respiratory factor 1, STAT6—signal transducer and activator of transcription 6, PGC-1α—peroxisome proliferator-activated receptor gamma coactivator, TLR4—toll-like receptor 4, VCAM-1—vascular cell adhesion molecule 1, ↑ - up-regulated, ↓- down-regulated.

## Abbreviations

PARP-1	poly (ADP-ribose) polymerase-1
PARPi	PARP inhibitors
AD	Alzheimer’s disease
PD	Parkinson’s disease
UV	ultraviolet light
ROS	reactive oxygen species overproduced in oxidative stress
RNS	reactive nitrogen species
TNFα	tumor necrosis factor α
iNOS	inducible isoform of nitrite oxide synthase
COX2	cyclooxygenase 2
MCP1	monocyte chemoattractantprotein 1
IL-1β, 6, 8	interleukins 1β, 6, 8
NF-κB	nuclear factor kappa-light-chain-enhancer of activated B cells
AP1, AP2	activator proteins 1 and 2
JAK	Janus kinase
PIP3	phosphatidylinositol (3,4,5)-trisphosphate
MAPKKKs	mitogen-activated protein (MAP) kinase kinase kinases
MAPKKs	mitogen-activated protein kinase kinases
MAPKs	mitogen-activated protein kinases
STAT	members of the signal transducer and activator of transcription protein family
AKT	subfamily of serine/threonine kinases
p38	p38 mitogen-activated protein kinases
JNK	c-Jun N-terminal kinases
ERKs	extracellular signal-regulated kinases
p300/CRBP	p300/CREB-binding protein complex
EMT	epithelial-mesenchymal transition
HSP70	heat shock protein 70
AIF	apoptosis inducing factor
HDAC	histone deacetylases
SIRT-1	sirtuin-1
AMPK	AMP-activated protein kinase
TNBS	trinitrobenzenesulfonic acid
PGC-1α	peroxisome proliferator-activated receptor gamma coactivator 1-α
TIMP-2	tissue inhibitor of metalloproteinases 2, I
ICAM-1	inter-cellular adhesion molecule 1
ALDH2	aldehyde dehydrogenase 2
TC	total cholesterol
VLDL	very low density lipoproteins
LDL	low density lipoproteins
ACAT1	acetyl-CoA acetyltransferase 1
SMC	smooth muscle cell content
CRP	C-reactive protein
MPO	myeloperoxidase
BRCA	BRCA1 (breast cancer type 1 susceptibility protein) and BRCA2 (breast cancer type 2 susceptibility protein)
LPS	lipopolysaccharide
MMPs	matrix metalloproteinases
MIP-1a and 2	macrophage inflammatory proteins 1a and 2
CXCLs	C-X-C motif chemokine ligands
GBP2	guanylate binding protein 2
IigP1	interferon-inducible GTPase 1
CD274	programmed death-ligand 1 or PD-L1
Gpx1,4	glutathione peroxidase 1, 4
SOD1	superoxidedismutase 1
mt-Nd1	mitochondrially encoded NADH
Sdha	succinate dehydrogenasecomplexflavoproteinsubunit A
mt-Cytb	mitochondrially encoded cytochrome B
FOXO1	forkhead box protein O1
Nrf1	nuclear respiratory factor 1
STAT6	signal transducer and activator of transcription 6
TLR4	toll-like receptor 4
VCAM-1	vascular cell adhesion molecule 1
QC	quercetin
RSV	resveratrol
NG	naringenin
GP	hesperetin
4MF	4’-methoxyflavone
DMF	3’, 4’-dimethoxyflavone
3-AB	3-aminobenzamide
TNK	tankyrases
TF	theaflavin polyphenol
EGCG	epigallocatechin gallate
